# Impact of differential DNA methylation on transgene expression in cotton *(Gossypium hirsutum L.)* events generated by targeted sequence insertion

**DOI:** 10.1111/pbi.13049

**Published:** 2019-01-19

**Authors:** Aurine Verkest, Stephane Bourout, Jurgen Debaveye, Kristine Reynaert, Bernadette Saey, Ilse Van den Brande, Kathleen D'Halluin

**Affiliations:** ^1^ BASF Agricultural Solutions Belgium NV Gent Belgium

**Keywords:** epigenome analyses, targeted sequence insertion, transgene silencing, DNA methylation, cotton, gene targeting, transgene expression, expression variability, differential silencing, DNA methylation mechanisms

## Abstract

Targeted Genome Optimization (TGO) using site‐specific nucleases to introduce a DNA double‐strand break (DSB) at a specific target locus has broadened the options available to breeders for generation and combination of multiple traits. The use of targeted DNA cleavage in combination with homologous recombination (HR)‐mediated repair, enabled the precise targeted insertion of additional trait genes (2m*epsps, hppd, axmi115*) at a pre‐existing transgenic locus in cotton. Here we describe the expression and epigenome analyses of cotton Targeted Sequence Insertion (TSI) events over generations. In a subset of events, we observed variability in the level of transgene (*hppd*,* axmi115*) expression between independent but genetically identical TSI events. Transgene expression could also be differential within single events and variable over generations. This expression variability and silencing occurred independently of the transgene sequence and could be attributed to DNA methylation that was further linked to different DNA methylation mechanisms. The trigger(s) of transgene DNA methylation remains elusive but we hypothesize that targeted DSB induction and repair could be a potential trigger for DNA methylation.

## Introduction

Targeted Sequence Insertion in cotton has been achieved by the introduction of a targeted DNA DSB and its repair by HR‐mediated insertion of trait genes. By using a customized meganuclease, we were able to precisely insert two herbicide tolerance (HT) genes (2m*epsps*,* hppd*) in close vicinity to a pre‐existing transgenic locus in cotton and demonstrated that the resulting molecular stack was transmitted as a single locus to further generations (D'Halluin *et al*., 2013). We have also used this approach to introduce a HT gene (2m*epsps*) combined with an insect control (IC) gene (*axmi115*) at the same pre‐existing transgenic locus in cotton. Originally, we selected the transgene integration position with the objective to limit transcriptional interference between the transgenes of the molecular stack and to obtain more predictable expression of the newly added transgenes at the pre‐existing good performing transgenic locus. Somewhat unexpectedly, we observed expression variability of the newly added *hppd* or *axmi115* genes in a subset of the TSI events.

It has already been reported that targeted insertion events in tobacco, generated by Cre‐lox mediated site‐specific transgene integration into a specific chromosomal location can produce alleles that express at a predictable level, as well as alleles that are differentially silenced while the alleles were identical at the DNA sequence level. Transcriptional gene silencing via DNA methylation was attributed as a trigger of the variation in transgene expression (Day *et al*., 2000).

DNA methylation is linked to regulation of gene expression, genomic imprinting, transposon silencing and chromatin structure in plants. In plants, methylation occurs at cytosines in CG, CHG and CHH contexts (where H is any nucleotide except G). It is established and maintained simultaneously by several DNA methyltransferases involved in distinct pathways. Establishment of plant DNA methylation in all sequence contexts is mediated by RNA‐directed DNA methylation (RdDM) (Law and Jacobsen, 2010; Matzke and Mosher, 2014). RdDM involves small interfering RNAs (siRNAs), which target the *de novo* DNA methyltransferase DOMAINS REARRANGED METHYLTRANSFERASE DRM2 to genomic sites for DNA methylation. Once established, DNA methylation is maintained by distinct DNA methyltransferases that are responsible for maintaining methylation at either CG, CHG or CHH sites (Law and Jacobsen, 2010; Stroud *et al*., 2014). Methylation maintenance at CG sites relies on DNA METHYLTRANSFERASE 1 (MET1). Maintenance of CHG methylation occurs through a self‐reinforcing loop that requires CHG methyltransferase CHROMOMETHYLASE 3 (CMT3) and histone 3 lysine 9 (H3K9) methyltransferases KRYPTONITE. Methylation at CHH sites occurs through two distinct mechanisms: H3K9me2 linked CHROMOMETHYLASE 2 (CMT2) or RdDM recruited DRM2. The functional consequences of DNA methylation on gene expression often depend on the location of the methylation relative to the gene. Many genes can tolerate substantial levels of methylation in flanking regions (Li *et al*., 2015a). The presence of gene body methylation seems to have minimal or no effect on gene expression (Bewick *et al*., 2016). By contrast, promoter and transcription start site methylation is often associated with gene silencing (Niederhuth *et al*., 2016).

To‐date, there are only a few publications describing targeted HR‐mediated transgene insertion in plants (Ainley *et al*., 2013; Begemann *et al*., 2017; Li *et al*., 2015b; Shukla *et al*., 2009; Svitashev *et al*., 2015). Furthermore, there is no information available about the transgene expression of identical TSI events over generations. In this study, we report on the characterization of transgene expression over generations. We observed variability over several generations in expression levels of the *hppd* or *axmi115* transgenes in a subset of TSI events with identical DNA sequence and this was observed over generations in both, independently generated but genetically identical events and between sister plants from the same event. Further analyses demonstrated that the variation of transgene expression is mediated by DNA methylation and suggest that the trigger(s) for silencing might engage different pathways.

## Results

### Cotton targeted sequence insertion events can show strong expression variation of the newly introduced transgenes

Using the customized COT‐5/6 meganuclease, we made targeted introduction of different transgene expression cassettes at a position located 2072 bp upstream of an existing cotton event that carries the *cry2Ae* and the *bar* genes (described in published patent application WO2008/151780). Besides the described homologous pCV211 donor DNA (D'Halluin *et al*., 2013) we used additional donor DNAs containing either the 2m*epsps*/*hppd* or the 2m*epsps*/*axmi115* expression cassettes flanked by cotton genomic sequences corresponding to the target locus. Details about the donor DNAs are listed in Table S1. The *hppd* and *axmi115* genes are referred to as the genes of interest (*GOI*) hereafter. The *hppd* gene conferring tolerance to 4‐hydroxyphenylpyruvate dioxygenase (*hppd*) inhibitors and the *axmi115* gene conferring insect control were each linked to the selectable marker (SM) gene, the double mutant enol‐pyruvylshikimate‐3‐phosphate synthase gene (2m*epsps*), allowing selection of plants on glyphosate. Co‐delivery, using particle bombardment, of the COT‐5/6 meganuclease gene and the respective donor DNAs into embryogenic callus (EC) of the target cotton line and consecutive selection of glyphosate tolerant EC events followed by PCR analysis allowed the recovery of targeted sequence insertion (TSI) events at frequencies ranging from 1.8 to 7.5% (Table S1). Southern blot analysis, sequencing of the PCR amplicon covering the recombination sites upstream and downstream of the insertion site and capture‐based target enrichment prior to Illumina Miseq next‐generation sequencing (NGS) allowed to identify TSI events with a homology‐directed insertion of 2m*epsps*/*hppd* or 2m*epsps/axmi115* at the target locus.

**Figure 1:**
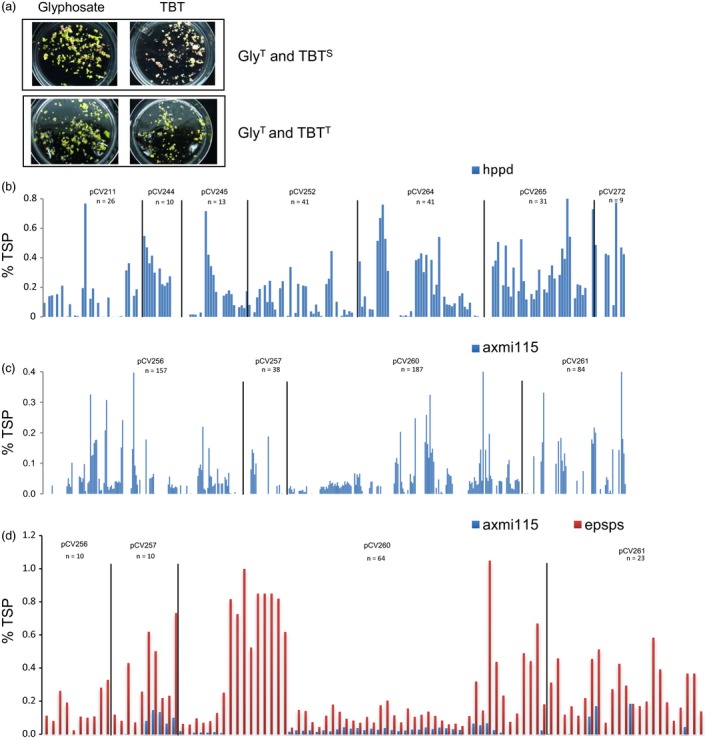
Targeted sequence insertion (TSI) events of different donor DNAs display variation in gene of interest (GOI) expression. (a) Sensitivity screening of embryogenic callus of glyphosate tolerant pCV211 events to the HPPD inhibitor herbicide tembotrione (TBT); TBT^S^, sensitive to TBT; TBT^T^, tolerant to TBT; Gly^T^, tolerant to glyphosate. (b) ELISA of HPPD protein expression in T0 plants, % HPPD of total protein is indicated (% TSP), *n* = number of plants analysed. (c) ELISA of AXMI115 protein expression in T0 plants, % AXMI115 of total protein is indicated (% TSP), *n* = number of plants analysed. (d). ELISA of AXMI115 and 2mEPSPS protein in a subset of T0 plants from Figure 1c. For each plant % 2mEPSPS (in red) and % AXMI115 (in blue; %TSP) is shown. pCV211, pCV244, pCV245, pCV252, pCV264, pCV265, pCV272 represent donor DNAs with a 2m*epsps*/*hppd* expression cassettes; pCV256, pCV257, pCV260, pCV261 represent donor DNAs with a 2m*epsps*/*axmi115* expression cassettes. Details about the donor DNAs can be found in Table S1.

For the recovery of glyphosate tolerant TSI events, we used the 2m*epsps* gene as selectable marker gene. In these glyphosate tolerant TSI events, we observed that the expression of the *hppd* or *axmi115* GOI in T0 plants from independent TSI events was variable (Figure 1, Table S2). Multiple T0 plants (sister plants) were regenerated from each independent EC event. With the 2m*epsps/hppd* TSI events, variability in expression of the *hppd* gene could already be observed in tissue culture. By plating EC of glyphosate tolerant events on substrate with the HPPD inhibitor herbicide tembotrione (TBT), events with only green, only white or both white and green embryos were observed (Figure 1a). Consistent with the observation of the *in vitro* TBT screen, ELISA analysis for HPPD protein expression in 169 T0 plants derived from 54 events, generated with seven donor DNAs, showed the presence of events where all plants express HPPD (‘positive’), events where all plants show no expression of HPPD (‘negative’), and events comprising both HPPD non‐expressing and expressing plants (‘mixed’) (Figure 1b, Table S2). This expression variability seemed to occur independently of the donor DNA sequence as for *axmi115*, similar observations with four donor DNAs were made. Protein expression analysis in 466 T0 plants originating from 126 events resulted in events where all plants express AXMI115 (‘positive’), events where all plants are silenced (‘negative’), or events with both AXMI115 silenced and AXMI115 expressing plants (‘mixed’) (Table S2, Figure 1c). In contrast, protein expression analysis by ELISA confirms expression of the 2m*epsps* gene which was expected as it was used as selectable marker gene for the recovery of glyphosate tolerant TSI events as shown for the AXMI115 TSI events (Figure 1d).

To identify for further downstream analyses clean TSI events devoid of any non‐targeted insertions of transforming DNA anywhere in the genome, we performed capture‐based target enrichment prior to Illumina MiSeq next‐generation sequencing (NGS) on genomic DNA isolated from several TSI events belonging to the different expression classes (positive, negative and mixed; Table S3). These clean TSI events displayed a HR‐mediated transgene insertion at the genomic cotton target site without additional random insertions of DNA from the donor DNAs and meganuclease vectors. Also, within these clean TSI events, we could identify events displaying variation or silencing of expression of the GOI (*hppd*/*axmi115)*. This variation in expression was not observed for 2m*epsps* which was not surprising as this gene was used as SM gene to select for glyphosate tolerant TSI events.

To summarize, these results show that TSI of 2m*epsps*/*hppd* or 2m*epsps*/*axmi115* at the same genomic location can give rise to genetically identical events that display variation in *hppd* or *axmi115* expression. Importantly, the variation in gene expression of the *hppd* or *axmi115* occurred independently of the expression of the linked 2m*epsps* SM gene. In addition, the observed variation in expression appeared to occur independently from the donor DNA sequence and expression cassette design since it was observed with different donor DNAs with the GOI under control of different promoters and the GOI in different orientations compared to the SM gene. The expression variation was not due to any epistatic effect of additional vector sequences integrated elsewhere in the genome.

### Clean TSI events show differences in GOI expression over generations

Progeny analysis was performed on a number of clean, independent TSI events. T1 progeny was generated most often through selfing and only through crossing with cotton line C312 for T0 plants with male sterility problems. T2 progeny and beyond were generated through selfing (see Table S4).

T1 up to T5 progeny were screened by real‐time PCR (Ingham *et al*., 2001) for copy number determination of the four transgenes – two from the TSI event (2m*epsps/hppd* or 2m*epsps/axmi115*), and two from the original cotton event (*cry2Ae*,* bar)* where the TSI were inserted. Hemizygous and homozygous progeny plants carried 1 and 2 copies, respectively, of each of the four genes of the molecular stack, while null segregants were null for all four genes. As reported previously, all T1 progeny did show inheritance of the molecular stack (*cry2Ae*/*bar*/*hppd* or *axmi115*/2m*epsps*) in a Mendelian manner as a single genetic locus (D'Halluin *et al*., 2013).

HPPD/AXMI115 protein expression was analysed by ELISA on multiple plants from independent TSI events over several generations (Table S4).

**Figure 2:**
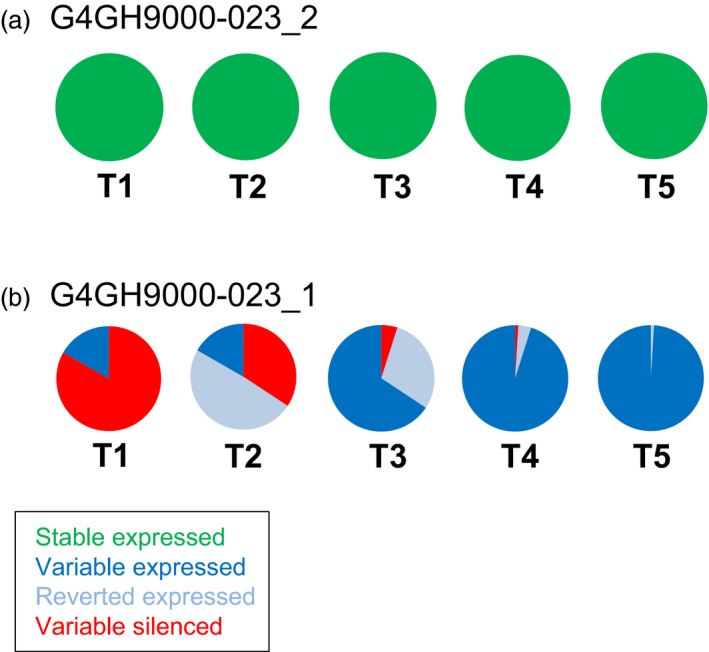
Gene of interest (GOI) expression is different between pCV211 sister plants and variable over generations. ELISA of HPPD protein expression over 5 generations (T1–T5) in TSI pCV211 G4GH9000‐023 event, sister plants G4GH9000‐023_2 (a) and G4GH900‐023_1 (b). Pie charts show the fraction of each expression class and the variation in expression over the generations. Green, ‘stable’ expressed; dark blue, ‘variable’ expressed; red, ‘variable’ silenced; pale blue, ‘reverted’ expressed. Plants analysed in each generation are indicated in Table S4. Donor DNA pCV211 contains a 2m*epsps*/*hppd* expression cassette (Table S1).

For the pCV211 donor DNA containing the *hppd* gene, progeny from 2 sister plants derived from the event G4GH9000_023 (G4GH9000‐023_1 and G4GH9000‐023_2), were followed from T1 through T5 generations. In the T1 generation all G4GH9000‐023_2 progeny plants expressed HPPD and the expression was maintained in 59 analysed plants up to generation T5 (‘stable’ expressed, green, Figure 2a). The T1 progeny of the sister plant G4GH9000‐023_1 displayed ‘variable’ expression of HPPD, with one plant displaying expression and five plants silencing. In later generations, the progeny of the expressing plant maintained HPPD expression (‘variable’ expressed, blue, Figure 2b). For T1 plants without HPPD expression, silencing was lost over generations. Starting with over 80% of silenced plants in T1 (‘variable’ silenced, red), 34% displayed silencing in T2, 5% in T3 and only 1% in T4. Finally, all progeny gained HPPD expression in T5 (Figure 2b). Progeny that reverted from non‐expressing progenitors (‘variable’ silenced, red) to expressing descendants (‘reverted’ expressed, pale blue) continued to stably express HPPD in subsequent generations (‘variable’ expressed, blue).

**Figure 3:**
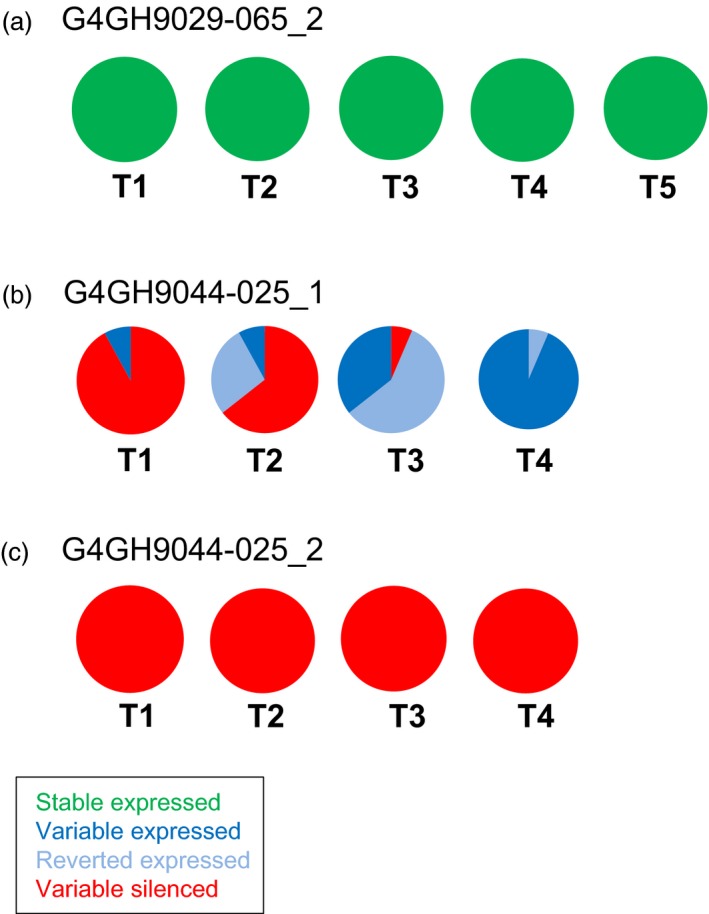
pCV260 *axmi115* GOI expression is differential among events, sister plants and generations. ELISA of AXMI115 protein expression in event G4GH9029‐065 (a), and event G4GH9044‐025, sister plants G4GH9044‐02502_1 (b) and G4GH9044‐02502_2 (c) over 4‐to‐5 generations (T1 to T4‐T5). Pie charts show the fraction of each expression class and the variation in expression over the generations. Green, ‘stable’ expressed; dark blue, ‘variable’ expressed; red, ‘variable’ silenced; pale blue, ‘reverted’ expressed. Plants analysed in each generation are indicated in Table S4. Donor DNA pCV260 contains a 2m*epsps*/*axmi* expression cassette (Table S1).

When looking at events generated with donor DNA pCV260 with the *axmi115* gene, several events were followed over maximum five generations (Table S4). Again, both stable (e.g. G4GH9029‐065, green, Figure 3a) and variable (e.g. G4GH9044‐025, Figure 3b and c) GOI expressing events were identified. In case of the variable AXMI115 expressing event G4GH9044‐025, for the plant G4GH9044‐02502_1, just as in the above example of the variable pCV211 G4GH9000‐023_1 plant, loss of silencing over generations could be observed (Figure 3b, Table S4). However, in its sister plant G4GH9044‐02502_2, silencing of AXMI115 was maintained and stable up to T4 (Figure 3c, Table S4).

**Figure 4:**
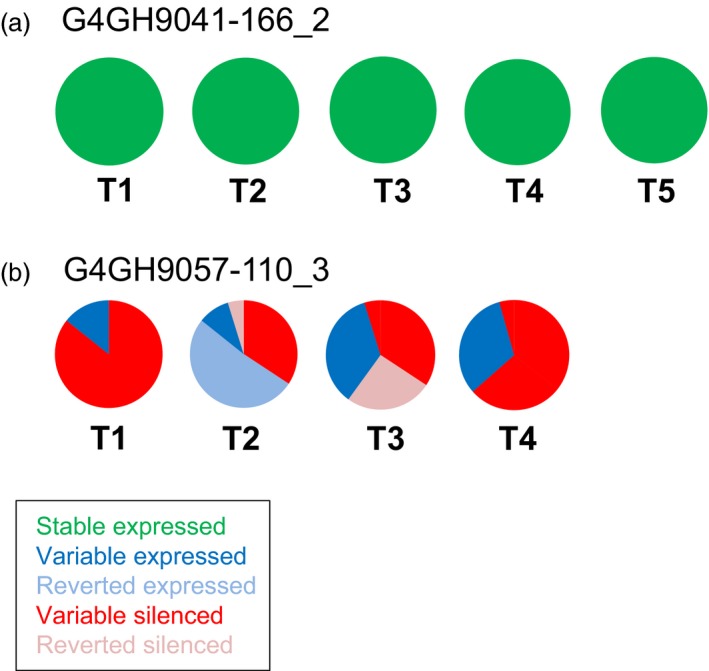
pCV261 *axmi115* GOI expression is differential among events and over generations. ELISA of AXMI115 protein expression in event G4GH9041‐166, sister plant G4GH9041‐166_2 (a) and event G4GH9057‐110, sister plant G4GH9057‐110_3 (b) over 4‐to‐5 generations (T1 to T4‐T5). Pie charts show the fraction of each expression class and the variation in expression over the generations. Green, ‘stable’ expressed; dark blue, ‘variable’ expressed; red, ‘variable’ silenced; pale blue, ‘reverted’ expressed; pink, ‘reverted’ silenced. Information on het number of plants analysed in each generation are indicated in Table S4. Donor DNA pCV261 contains a 2m*epsps*/*axmi* expression cassette (Table S1).

Finally, *axmi*115 donor DNA pCV261 progeny from two different TSI events were analysed (Table S4). Stable AXMI115 expression was observed in all 35 pCV261 G4GH9041‐166_2 progeny plants up to T5 (Figure 4a, green). In the event G4GH9057‐110, different expression patterns were seen within the progeny of plant G4GH9057‐110_3 (Figure 4b, Table S4). Some T1 progeny plants displaying AXMI115 expression (‘variable’ expressed, blue) gave rise to silenced progeny (‘reverted’ silenced, pink) and this established AXMI115 silencing remained stable (‘variable’ silenced, red) in its descendants while other T1 plants displaying AXMI115 silencing (‘variable’ silenced, red), showed partial loss of silencing in their progeny with 60% expressing descendants in T2 (‘reverted’ expressed, pale blue). In T3 re‐establishment of AXMI115 silencing was seen in 50% of the progeny plants (‘reverted’ silenced, pink) of a reverted expressing T2 plant, and this silencing was maintained in the T4 progeny (‘variable’ silenced, red; Figure 4b, Table S4).

Quantitative RT‐PCR was performed on several plants to confirm the ELISA results. GOI stable expressing (green), variable expressing (blue) and variable silenced (red) plants were analysed in different generations (Figure S1; Table S4). GOI RT‐qPCR results were in line with the ELISA results and confirmed the variable GOI expression and silencing within some events (Figure S1). In contrast, examination of the expression level of the 2m*epsps* SM gene, corroborated the glyphosate tolerance selection and was relatively steady‐state. Also, analysis of the mRNA levels of transgenes from the original cotton event (*bar* and *cry2Ae*), demonstrated stable and comparable expression in all tested lines (Figure S1).

In summary, the analysed clean TSI events could be grouped in two general categories: ‘stable’ events for which all plants show GOI expression over generations, and ‘negative’ or ‘mixed’ events, that show variability in GOI expression. The latter display different outcomes of GOI expression in their progeny with GOI expression remaining stable, or being lost or gained over generations. Remarkably, sister plants of the same event sometimes show a very different expression pattern.

### GOI expression variability is associated with differences in promoter and coding sequence DNA methlylation levels

To determine why *hppd/axmi115* expression levels differ between and/or within independent TSI events, and vary over generations, we quantified DNA methylation levels of the inserted GOI sequences by targeted bisulfite sequencing of a subset of plants from selected expressing and variable TSI events of different donor DNAs (Table S4).

**Figure 5:**
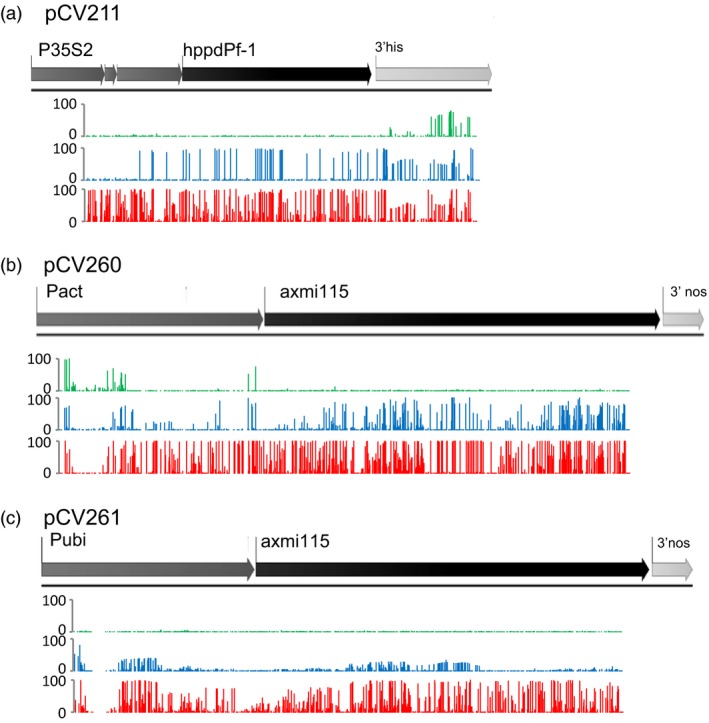
Variable GOI expression is associated with DNA methylation. Targeted bisulfite sequencing on progeny plants of stable expressing (green), variable expressing (blue) and silenced (red) plants. Plants originating from the events in Figures 2, 3 and 4 were analysed by targeted bisulfite sequencing (see Table S4). (a) pCV211 donor DNA, plants G4GH9000‐23_2 (green) and G4GH9000‐023_1 (blue and red). (b) pCV260 donor DNA, plants G4GH9029‐065_2 (green), G4GH9044‐025_1 (blue) and G4GH9044‐025_2 (red). (c) pCV261 donor DNA, plants G4GH9041‐166_2 (green) and G4GH9057‐110_3 (blue and red). Mean methylation density per cytosine in all contexts is plotted on a 0–100% scale. Analyses on additional samples is shown in Figure S2. pCV211 donor DNA contains a 2m*epsps*/*hppd* expression cassette; pCV260 and pCV261 represent donor DNA with a 2m*epsps*/*axmi115* expression cassette (Table S1).

In pCV211 event G4GH9000‐023 (Figure 5a) we saw differences in DNA methylation of the GOI between plants stably expressing *hppd* (G4GH9000‐023_2, green) and plants whose progeny displayed variable expression (G4GH9000‐023_1, blue and red). In the stably expressing plants (green) no GOI methylation was observed outside the 3′ UTR sequence. In the variable expressing (blue) plants methylation was restricted to the coding and 3′ UTR sequence while in the silenced (red) plants methylation spread along the coding sequence and the promoter (Figure 5a). The consistency of the DNA methylation distribution pattern within the three expression classes (stably expressed GOI, variable expressed GOI and silenced GOI) was confirmed in different plants over two generations (Figure S2A). Hypermethylation of the promoter was specific for plants silenced (red) for the GOI and reduced in progeny of silenced plants with regained *hppd* expression (reverted expressed, pale blue; Figure S2A).

In pCV260 TSI plants from a stable expressing event (G4GH9029‐065, Figures 5b and S2B, green) limited GOI DNA methylation was observed in the promoter and the region close to the transcription start site. The pCV260 G4GH9044‐025_1 variable expressing plants displayed DNA methylation in almost the entire coding sequence and discrete parts of the promoter in progeny showing *axmi115* expression (Figures 5b and S2B, blue). In plants silenced for *axmi115* expression, the DNA methylation pattern became denser and covered the complete GOI coding sequence and its upstream promoter region (Figures 5b and S2B, red).

Similarly, stable expressing pCV261 G4GH9041‐166_2 progeny (green) appeared to have a hypomethylated (no DNA methylation) GOI sequence. Progeny of the ‘mixed’ pCV261 G4GH9057‐110 event that express *axmi115* (blue) showed weak methylation in regions of both the promoter and *axmi115* coding sequence that became stronger and spread along the coding sequence and promoter in silenced (red) plants (Figures 5c and S2C).

Together these results reveal different methylation patterns between expressing and silenced plants showing a clear inverse correlation between DNA methylation and GOI expression levels. Irrespective of the inserted transgenes, stable expressing plants displayed no or little methylation whereas silencing appeared linked with heavy promoter and CDS hypermethylation. In variable expressing plants, the coding sequence (CDS) methylation shows GOI sequence specific distribution patterns.

**Figure 6:**
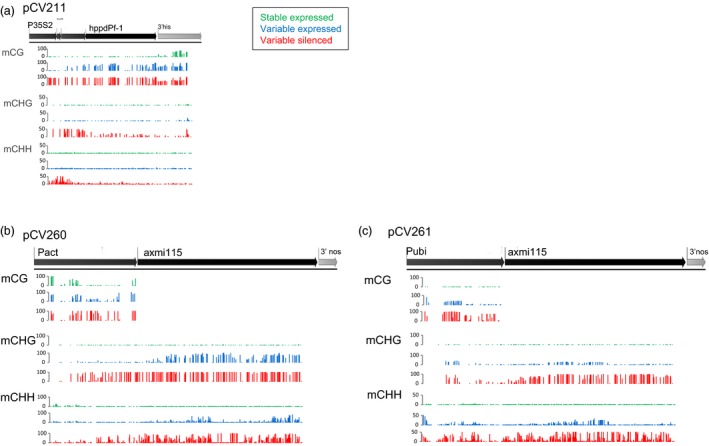
CG, CHG and CHH context methylation analysis. Context specific methylation rates within the GOI in (a) pCV211, (b) pCV260 and (c) pCV261 donor DNA TSI events were determined by bisulfite methylation sequencing. Mean methylation density per cytosine context is plotted to the GOI on a 0–100% or 0–50% scale. Green, ‘stable’ expressed; blue, ‘variable’ expressed; red, ‘variable’ silenced. Plants analysed were the same as shown in Figure 5 (see Table S4). pCV211 donor DNA contains a 2m*epsps*/*hppd* expression cassette; pCV260 and pCV261 represent donor DNA with a 2m*epsps*/*axmi115* expression cassette (Table S1).

To get more insight in the establishment and role of the DNA methylation signatures we looked at the methylation sequence contexts (Figures 6 and S3). Inspection of the methylation density in the CG, CHG and CHH contexts revealed specific differences among the different donor DNA events. For pCV211, the variable expressing progeny (blue) of plant G4GH9000‐023_1 shows *hppd* gene body methylation in the CG context. In *hppd* silenced plants (red) CG, CHG and CHH gene body and promoter methylation is accumulating (Figures 6 and S3A). For pCV260 and pCV261 events gene body CG methylation cannot occur since the *axmi115* CDS has no CGs. pCV260 variable expressing plants (blue) are mainly characterized by CHG CDS methylation and CG promoter methylation. Silencing requires additional promoter CG, CHG and CHH, and CDS CHG and CHH sequence methylation (Figures 6 and S3B, red). *Axmi115* variable expressing (blue) pCV261 plants show different methylation patterns than pCV260 plants and display mainly promoter CG, CHG and CHH methylation. In silenced (red) pCV261 plants, promoter methylation intensifies and emerges/arises within the CDS (Figures 6 and S3C).

Together, the DNA methylation data showed transgene silencing being correlated with GOI promoter, transcription start site (TSS) and 5′ coding sequence (CDS) DNA methylation with differences in methylation contexts. Although these differences are likely GOI sequence context dependent, this indicates that alternative pathways might induce and maintain the methylation dependent variable transgene expression and stability in TSI events.

### Variable contribution of sRNA and histone marks to GOI silencing

**Figure 7:**
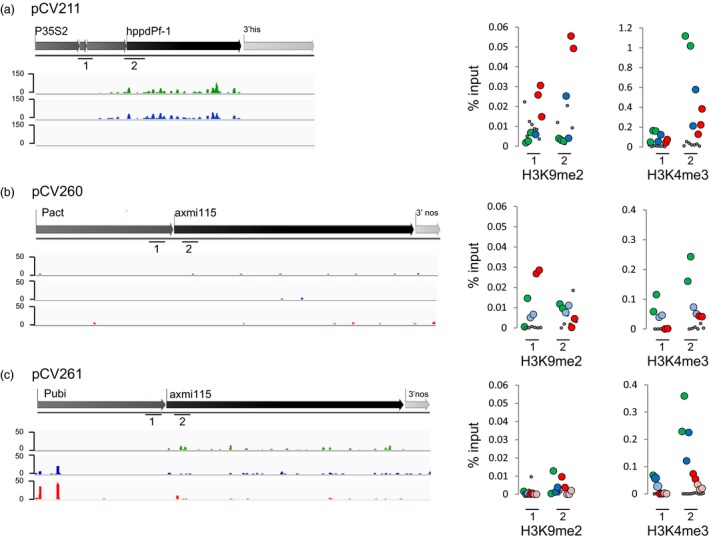
Variable transgene expressing TSI plants have different 24‐nt sRNA and histone mark GOI accumulation patterns. sRNA sequencing and ChIP‐qPCR on samples of stable expressing, variable expressing and silenced plants. Green, ‘stable’ expressed; dark blue, ‘variable’ expressed; red, ‘variable’ silenced; pale blue, ‘reverted’ expressed; pink, ‘reverted’ silenced. (a) pCV211 donor DNA, (b) pCV260 donor DNA and (c) pCV261 donor DNA TSI plants. Left, Mapping of the 24‐nt sRNA sequencing reads to the GOI sequences. The *y* axes are scaled the same per TSI/GOI. Reads were normalized per 10 million of genome‐matched (including the transgene sequence) 18‐to‐28 nucleotide sequences. Analyses on additional samples and accumulation of other sRNA size classes on the GOI (*hppd*/*axmi*) and complete TSI (*hppd*/*axmi* + *epsps*) sequence are shown in Figures [Link], [Link]–S6. Right, H3K9me2 and H3K4me3 ChIP. Enrichment was determined by qPCR and for each region normalized against the input. Analysed regions are indicated with a black line with digit 1 and 2 below it. Dots represent biological replicates. We indicate in Table S4 which plants have been analysed (ChIP column). Grey, background mock ChIP values.

To infer the molecular mechanisms responsible for DNA methylation in the different TSI events, we looked whether the GOI sequences are associated with small RNAs (sRNA). sRNAs, in particular 24‐nt sized sRNAs, are a hallmark of RdDM all context *de novo* and CHH maintenance DNA methylation (Law and Jacobsen, 2010). Illumina Hiseq of sRNAs was performed on stable expressing, variable expressing and silenced plants generated from TSI events of different donor DNAs. Profiling of the sRNAs mapping to the different TSIs was performed and their distribution and abundance on both the complete transgene insert and the GOI in stable expressing (green), variable expressing (blue) and silenced (red) plants was investigated (Figures 7 and [Link], [Link]–S6). Accumulation of GOI associated 24‐nt sRNAs in silenced plants would indicate RdDM mediated methylation.

For the pCV211 TSI event no accumulation of *hppd* associated 24‐nt sRNAs is seen in silenced plants (Figures 7a and S4). In contrast, we observed higher levels of *hppd* mapping sRNAs in expressing plants (see Discussion). Besides 24‐nt sRNAs, also 21‐nt GOI targeted sRNAs accumulate in expressing plants suggesting post‐transcriptional regulation of gene expression (Figure S4).

In pCV260 TSI plants, very few sRNAs target the GOI and no evidence points to RdDM mediated silencing (Figures 7b and S5).

Finally, pCV261 TSI plants also displayed a low number of sRNAs dispatched along the GOI and the complete TSI sequence. However, a discrete accumulation of sRNAs targeting the *axmi* 115 expression driving promoter is observed in variable expressing (blue) and silenced (red) plants (Figures 7c and S6). Whether these potentially initiate methylation through RdDM remains to be analysed.

Next, we examined distribution of H3K9me2 a hallmark of heterochromatin, associated with CHG and CHH methylation by CMT and DRM DNA methyltransferases (Law and Jacobsen, 2010). Also, H3K4me3 accumulation, a mark linked with active transcription, was tested. Chromatin immunoprecipitation (ChIP) and qPCR of promoter, TSS and/or 5′CDS regions of the GOI, associated with differential DNA methylation in expressing and silenced plants, was performed.

In pCV211 TSI events, accumulation of the repressive heterochromatin H3K9me2 mark was observed in silenced (red) plants in the regions comprising the promoter and the 5′ CDS of the *hppd* gene (Figure 7a). In contrast, stable *hppd* expressing (green) plants failed to accumulate H3K9me2 in these regions but displayed accumulation of the transcription linked H3K4me3 mark at the *hppd* 5′CDS (Figure 7a).

*Axmi115* silencing (red) in pCV260 TSI plants was found to be associated with an enriched deposition of repressive H3K9me2 in the promoter region just upstream of the TSS, whereas stable *axmi115* expressors (green) accumulate H3K4me3 in their 5′ CDS region (Figure 7b).

This higher H3K4me3 accumulation in the *axmi115* 5′ CDS sequence was also observed in stable pCV261 TSI *axmi115* expressing plants (green). However, pCV261 silenced plants (red) did not display enriched heterochromatin H3K9me2 deposition in the analysed promoter and TSS regions (Figure 7c).

Combined, these results illustrate that the contribution of sRNAs and H3K9me2 to GOI silencing appears variable, strengthening the variable DNA methylation context data and suggesting that alternative pathways trigger and/or maintain DNA methylation of the transgenes in the different TSI events.

## Discussion

In this study, we analysed the transgene expression of a number of cotton plants generated from independent TSI events or derived from sister plants of the same TSI event. TGO based on the use of site‐specific nucleases, combined with the increasing insight into genome sequences and gene functions, is expected to facilitate insertion of (trait) transgenes at predetermined integration sites, so called ‘safe harbours’. Targeted integration is perceived as advantageous over random integration because it could allow for the desired expression of the GOI while reducing or eliminating possible unintended effects due to disruption of native genes and regulatory elements associated with random transgene integration. However, site‐specific integration of (trait) transgenes by HR in plants has been rarely reported and currently there is no information available on the stability/variability of gene expression from such targeted integration events.

Here, analysis of cotton TSI events generated via HR, demonstrated that for a subset of the events, considerable variation in GOI (*hppd* or *axmi115*) expression over generations exists between plants generated from independent TSI events and between sister plants derived of the same TSI event. Whereas variation in transgene expression between random integration events has been described frequently, and laid the basis of epigenetic research (Meyer, 2013), reports on targeted integration events are scarce. Day *et al*. (2000) described the cre‐lox mediated generation of targeted transgene integrated tobacco events and attributed DNA methylation as a trigger of observed variable transgene expression. Using a mechanistically different approach for targeted integration of transgenes, also we identified transcriptional gene silencing through differential DNA methylation of the transgene promoter as at least one of the factors mediating variation in GOI expression in the analysed TSI events. Even gene targeting based on gene replacement for the targeted introduction of a few amino acid substitutions in an endogene could lead to changes in the DNA methylation profile as has been shown in *Arabidopsis* (Lieberman‐Lazarovich *et al*., 2013). Methylation could be either completely lost in a region of the target gene, maintained with minor changes, or show variability in subsequent generations.

Transcriptional gene silencing can be affected by various factors. These include characteristics of the insert, the position of and nature of the integration site and plant developmental stages and/or environmental conditions. Our experimental strategy and the obtained observations exclude several well‐established causes implicated in gene silencing.

As we observed variable GOI expression in clean TSI, we can eliminate the possibility of homology‐dependent transgene silencing (HDGS) due to integration of small stretches of donor or vector backbone DNA (Selker, 1999).

Also the occurrence of silencing triggered by the donor DNA sequence itself, is unlikely as variable transgene expression was observed in TSI events generated by using donor DNAs with different construct designs, promoters and GOIs. In case of the *hppd* GOI events, it is unlikely that the endogenous native *hppd* gene might trigger silencing of the *hppd* transgene as the level of DNA sequence homology between both genes is less than 40%. In TSI events where the GOI is under the control of the 35S promoter that also drives the expression of the cry2Ae gene from the original cotton event, the double presence of the 35S promoter might have influenced the level of GOI expression in some events. Especially since it has been reported that the 35S sequence is known to be susceptible to silencing by methylation (Okumura *et al*., 2016; Wang *et al*., 2017). However, we believe that another, more general trigger is at play since we observed silencing in TSI events with other GOI promoters while the expression of the 35S driven cry2Ae gene was stable and not affected in any of the plants tested.

Several reports describe a correlation between the incidence of gene silencing and high transgene copy number, but here single copy TSI events were selected and confirmed by Illumina MiSeq next‐generation sequencing (NGS) after capture‐based target enrichment, not to contain additional random insertions of DNA from the donor DNAs and meganuclease vectors.

Similarly silencing originating from the allelic state of the sequence insertion (Masclaux *et al*., 2005) can be ruled out since no correlation was seen between GOI expression variability/stability in selfed, backcrossed, hemizygous or homozygous TSI progeny.

With regard to the integration site, position effects frequently have been associated with variable expression (Matzke and Matzke, 1998) although some studies suggest that these play minor roles (Nagaya *et al*., 2005). Nevertheless, transgene integration into heterochromatic regions has been shown to lead to silencing (Ahmed *et al*., 2011; Hollister and Gaut, 2009). Here, targeted integration was done at an approximately 2000 bp distance from the locus of an existing cotton event which is positioned in a gene‐rich genomic region without interrupting any functional annotated sequences.

Also, we have to take in account that cotton transformation is particularly challenging because of its strong genotype dependence for regeneration through somatic embryogenesis, its long timelines and its high level of somaclonal variation (Kumria *et al*., 2003). The duration of the tissue culture period including cell dedifferentiation and differentiation processes contributes to enhancing the rate of somaclonal variation and changes in DNA methylation. This process of tissue culture and regeneration was reported to induce consistent and stable epigenomic changes in both rice and maize (Stelpflug *et al*., 2014; Stroud *et al*., 2013a). However, we observed high variability in GOI expression and methylation between independent TSI events, between sister plants of the same TSI event, and this over generations making somaclonal variation an unlikely main trigger of expression variability observed for *hppd* and *axmi115* genes but not for the three other transgenes (*cry2Ae*,* bar* and 2m*epsps*).

Regardless of the donor DNA, all silenced events were characterized by promoter and transcription start site (TSS) DNA methylation. Promoter and TSS methylation is often associated with gene silencing (Niederhuth *et al*., 2016). Similarly, stable expressing events had almost no methylation in both the promoter and coding sequence (CDS). In contrast, the different variable expressing TSI events displayed different methylation context and distribution patterns. Part of these could be attributed to GOI sequence differences. For example, all *axmi115* TSI events, in contrast to *hppd* events, are devoid of CG gene body methylation since the *axmi115* CDS sequence has no CGs. However, other differences appeared to be GOI sequence independent. *Axmi115* CDS methylation is different in pCV260 and pCV261 variable expressing plants. Also different context methylation of the promoters in variable expressing plants was observed, despite the presence of all sequence contexts in these different sequences. This suggests that different methylation pathways induce and maintain the transgene silencing in different TSI events. Substantial methylation in all three contexts (CG, CHG and CHH) may result from RdDM and/or CMT2 (Stroud *et al*., 2014; Zemach *et al*., 2013). Regions with CG or CHG methylation that do not have CHH methylation probably are maintained by MET1 and CMT3 (Bewick *et al*., 2016; Stroud *et al*., 2013b).

sRNA‐sequencing revealed no accumulation of RdDM associated 24‐nt promoter targeting sRNAs in the GOI silenced pCV211 and pCV260 TSI plants. For pCV261 TSI events a discrete accumulation of two ubiquitin promoter mapping 24‐nt sRNAs was seen in variable expressing and silenced plants. Whether these are triggering variability in *axmi115* expression remains to be explored. The bisulphite‐sequencing data illustrated relatively dense cytosine methylation in the pCV261 GOI promoter region targeted by these sRNAs. Also, mainly the altered methylation of regions, and not positions, has documented effects (Cubas *et al*., 1999; Manning *et al*., 2006; Ong‐Abdullah *et al*., 2015). Somewhat unexpectedly, given their established role in RdDM, we observed accumulation of 24‐nt sRNAs mapping to *hppd* in pCV211 TSI expressing plants. Recently, various noncanonical RdDM mechanisms have been described (Cuerda‐Gil and Slotkin, 2016) illustrating exceptions on the classical biogenesis pathways and size classification of 24‐nt sRNAs for RdDM mediated TGS and 21‐nt sRNAs for PTGS. An example is 21‐nt sRNAs guiding RdDM (Marí‐Ordóñez *et al*., 2013; Nuthikattu *et al*., 2013). Conversely, 24‐nt sRNAs have been reported to mediate post‐transcriptional gene silencing (Klein‐Cosson *et al*., 2015). Interestingly, besides 24‐nt sRNAs, also 21‐nt *hppd* mapping sRNAs accumulate in GOI expressing plants. As both sRNA populations display a similar accumulation pattern these might share the same dsRNA precursors. Additional experiments would be needed to address the role of both 24‐nt and 21‐nt siRNA accumulation in pCV211 transgene expressing plants.

Also in *C. elegans* variable expression was observed for transgenes inserted into the same locus by homologous recombination. They found a class of small RNAs (piRNAs) involved in silencing of foreign RNA sequences. Initiation of silencing involves the comparison of the foreign sequence to an epigenetic memory of previously expressed sequences. Maintenance of silencing requires chromatin factors and RdRP‐generated small RNAs. Activating and silencing signals may compete in foreign versus non‐foreign discrimination. Thus also in C.elegans genetically identical targeted insertion individuals can show remarkably opposite patterns of expression, very similar to what we have observed in cotton TSI events although epigenetic pathways may differ widely from *C. elegans* to plants (Seth *et al*., 2013; Shirayama *et al*., 2012).

The interplay of H3K9me2 accumulation with CHG and CHH DNA methylation in silenced plants was confirmed in pCV211 and pCV260, but not pCV261 events. This, in combination with the absence of massive GOI targeting by sRNAs in silenced plants, indicates an independence of the RdDM pathway. Also it suggests maintenance of silencing via different pathways in the different donor DNA TSI events. Maybe RdDM *de novo* DNA methylation was established in the T0 generation and only maintenance methylation pathways are acting in analysed progeny. This could explain part of the observed variability in GOI expression, namely stable silencing and loss of silencing over generations, but cannot account for re‐established silencing in later generations. However, we cannot exclude the possibility that this *de novo* silencing, which was only observed in pCV261 events, is an additional distinct co‐occurring silencing mechanism specific for these lines. Different silencing pathways often co‐occur, confounding the interpretation of their mechanisms. Alternatively, *de novo* methylation might be induced by another mechanism utilizing the different DNA methyltransferases independently of RdDM (Ahmed *et al*., 2011; Singh *et al*., 2008).

Looking at open chromatin regions (using DNaseI, ATAC‐seq or NicE‐seq techniques) between silenced and expressed transgenes could shed additional light on the mechanism leading to gene expression or repression (Chandler and Vaucheret, 2001; Ponnaluri *et al*., 2017; Wang *et al*., 2018).

The trigger responsible for variable silencing in different independent clean TSI events as such remains elusive. An intriguing possibility lies in the targeted DSB induction. Studies in mammalian cells have shown that targeted DNA methylation occurs upon homology‐mediated repair of induced DSBs, resulting in variable gene expression among cells (Cuozzo *et al*., 2007; Morano *et al*., 2014; Russo *et al*., 2016). This is facilitated through recruitment of DNA methyltransferases and histone‐modifying enzymes at the DSB during HR. Upon targeted DSB induction, the transcribed strand blocked by stalled RNA PolII may become preferential target for DNA methyltransferases and other proteins that regulate methylation (Morano *et al*., 2014). Whether this also occurs in plant cells is not known. A role for the DDB2 DNA damage factor in DNA methylation through both RdDM and regulation of DNA methylcytosine glycosylase has been shown in *Arabidopsis* (Schalk *et al*., 2016). Also, interconnections between sRNAs and DNA damage repair have been reported (Schalk *et al*., 2017; Wei *et al*., 2012). DSBs were shown to trigger the induction of sRNAs required for mediating RdDM independent DSB repair (Wei *et al*., 2012). A link between DNA DSB repair and DNA methylation in plants remains to be further investigated and should give interesting insights, particularly for TGO applications.

The fact that a considerable variation in GOI (*hppd*, or *axmi115*) expression was observed in a subset of clean TSI events shows that even when integration events are targeted, selection remains necessary similarly to the practice for random integration events in order to identify TSI events with stable GOI expression over generations.

## Experimental procedures

### Plant material

Targeted Sequence Insertion (TSI) plants were grown in controlled environment greenhouse compartments with a 16 h light period at 26 °C under lighting and 20 °C during darkness and 40%–60% relative humidity. Leaf material at the same developmental stage (2nd to 5th fully expanded leaf) was harvested from 5 to 6‐week‐old seedlings. Targeted insertion events with the *hppd,* 2m*epsps* or *axmi,* 2m*epsps* genes were generated in an existing cotton event that carries the *cry2Ae* and the *bar* genes, conferring Lepidoptera resistance and glufosinate tolerance, respectively. The *cry2Ae* gene was cloned between the 35S promoter and the 3′35S terminator and the *bar* gene between the Csvmv promoter and the 3′nos terminator. The procedure for generation of targeted insertion events in cotton is described in (D'Halluin *et al*., 2013). The design of the repair DNAs is shown in Table S1.

### RNA/DNA analyses

Genomic DNA was isolated using a DNeasy Plant Mini kit (Qiagen, Hilden, Germany). RNA was extracted with a Spectrum Plant Total RNA kit (Sigma, Saint‐Louis, MO).

All qPCRs were performed with the Fast SYBR Green Master Mix (Thermo Fisher Scientific, Waltham, MA) and amplified on a Bio‐Rad CFX 384.

For RT‐qPCR, RNA was treated with HL‐dsDNase (ArticZymes) and reverse transcribed with a High‐Capacity cDNA Reverse Transcription kit (Thermo Fisher Scientific). qPCR results were normalized to Gh_pp2a1 (Artico *et al*., 2010) and are expressed as 2^‐(Cq target‐Cq Gh_pp2a1)^.

### Protein analyses

The levels of 2mEPSPS, PAT, cry2Ae and HPPD protein were quantified using an enzyme‐linked immunosorbent assay (ELISA) using Envirologix kits with Envirologix‐catn° AP 084 NW V10, AP013, AP005CTNW V10 and AP‐126 NWV10, respectively. All quantitations were normalized to the protein concentration of the cotton leaf extract, as determined using the Coomasie Protein Assay Reagent Kit from Biorad.

### Capture‐based target enrichment and Illumina MiSeq next‐generation sequencing (NGS)

Targeted sequence capture of cotton DNA samples and sequencing on Illumina MiSeq was done by Eurofins Genomics (https://www.eurofins.com). DNA probe library design and preparation, targeted capture, sequencing and data analysis were done as described in Kovalic *et al*. (2012) and Shearer *et al*. (2012).

### Targeted Bisulfite sequencing

Bisulfite conversion, PCR‐based library generation, sequencing and analysis were performed by Active Motif (http://www.activemotif.com). Reads were aligned to the transgene sequences using the *bismark* software. Alignment and methylation information was captured in BAM files, and CpG alignment coverage and percentage methylation at each C site was determined. The alignment and cytosine methylation details are shown in Table S5.

### sRNA sequencing analysis

Custom libraries for sRNAs were constructed and sequenced by Fasteris SA (http://www.fasteris.com/). Small RNA reads were filtered to 18‐to‐28 nt reads and aligned to the *Gossypium hirsutum* genome (Li *et al*., 2015c) with the transgene DNA integrated at the targeted insertion. The following *bowtie* (version 0.12.9) parameters were used: (‐a, report all alignments per read; ‐v 1, report end‐to‐end hits with <=1 mismatch; –best hits, guaranteed best stratum; –strata, hits in sub‐optimal strata aren't reported). Reads were normalized to reads per million (RPM) of mapped reads. For visualization of transgene DNA directed sRNAs 20‐to‐22‐ and 23‐to‐25‐nt sRNAs reads were size extracted.

### ChIP‐qPCR

Chromatin immunoprecipitation was performed as described by Luo *et al*. (2013) with some minor changes.

0.5 g ground frozen cotton leaf material was suspended in 45 mL nuclei isolation buffer I (10 mm Hepes pH=7.6, 440 mm sucrose, 5 mm KCl, 5 mm MgCl2, 5 mm EDTA, 1% formaldehyde, 0.1% β‐mercapto‐ethanol, 0.5% Triton X‐100, 0.4 mm PMSF, Complete EDTA‐free Protease Inhibitor Cocktail (1/50 mL) (Sigma‐Aldrich, St Louis)) and incubated for 5 min at room temperature. Glycine was added to a final concentration of 125 mm and incubated at room temperature for 5 min to stop the crosslinking. The lysate was filtered twice over a 70‐um nylon mesh (Fisher Scientific). Nuclei were pelleted by centrifugation at 2000 × ***g*** for 10 min at 4 °C and the pellet was suspended with 3 mL of nuclear isolation buffer II (10 mm Hepes pH=7.6, 250 mm sucrose, 5 mm KCl, 5 mm MgCl2, 5 mm EDTA, 0.1% β‐mercapto‐ethanol, Complete EDTA‐free Protease Inhibitor Cocktail). The nuclei suspension was put onto a 30% Percoll nuclear isolation buffer II and centrifuged at 1500 ×  ***g*** for 20 min at 4 °C. The nuclei pellet was dissolved in 2 mL nuclei lysis buffer (50 mm Tris‐Cl pH 7.5, 0.1% SDS, 10 mm EDTA, 50 mm NaCl, Complete EDTA‐free Protease Inhibitor Cocktail), incubated at 4 °C during 1 h. The chromatin was sheared to 100–300 bp using milliTUBE 1 ml AFA Fiber tubes with a CovarisM220 sonicator (Covaris Inc., Brighton, UK).

Fragmented chromatin samples were cleared by centrifugation at 2000 × ***g*** for 5 min at 4 °C before being diluted in an equal volume of ChIP dilution buffer (50 mm Tris‐Cl pH 7.5, 0.2% Triton X‐100, 50 mm NaCl, 0.1 mm PMSF, Complete EDTA‐free Protease Inhibitor Cocktail). 20 μL of Dynabeads Protein G (Invitrogen, Thermo Fisher Scientific, Waltham, MA) were washed twice and resuspended in 100 μL of Incubation buffer (20 mm Tris pH 7.5, 50 mm NaCl, 5 mm EDTA, 0.1% TritonX). Antibodies were added: H3K4me3 (1 μg of Millipore 07‐473, http://www.merckmillipore.com/) or H3K9me2 (2 μg of Millipore 07‐441) and incubated for 2 h at 4 °C with gentle rotation on a wheel. Beads were washed in Incubation buffer and loaded with 200 μL (H3K4me3) or 400 μL (mock and H3K9me2) diluted chromatin solution that was pre‐cleared with 20 μL Dynabeads Protein G for 2 h at 4 °C. The chromatin/antibody/bead mix was incubated overnight at 4 °C with rotation. Beads were washed with 500 μL Incubation Buffer and 500 μL Wash Buffer (50 mm Tris‐Cl pH 7.5, 10 mm EDTA) with 50 mm (Wash1), 100 mm (Wash2) and 150 mm NaCl (Wash3), subsequently. Final wash was done in TE buffer (twice). The immunocomplexes were eluted twice with 150 μL of 1% SDS, 0.1 m NaHCO3 at 65 °C for 10 min. 12 μL 5 m NaCl was added and samples were incubated overnight at 65 °C. Samples were digested with 20 μg of proteinase K for 2 h at 42 °C followed with phenol/chloroform/IAA extraction and MinElute DNA purification (Qiagen) of the aqueous phase to obtain qPCR ready DNA. Results are expressed as % input.

## Supporting information


**Figure S1** RT‐qPCR in GOI stable expressing, variable expressing and silenced pCV211, pCV260, and pCV261 plants.

**Figure S2** Targeted bisulfite sequencing.

**Figure S3** Different methylation contexts invoke unstable or silenced expression in different TSI events.

**Figure S4** Results sRNA sequencing from pCV211 TSI plants.

**Figure S5** Results sRNA sequencing from pCV260 TSI plants.

**Figure S6** Results sRNA sequencing from pCV261 TSI plants.

**Table S1** List of donor DNAs and obtained TSI frequencies.
**Table S2** Overview of ELISA on T0 plants.
**Table S3** Summary of targeted DNA sequencing results.
**Table S4** List of all TSI event plants/progeny analysed.
**Table S5** Bismark targeted DNA methylation sequencing reports.
