# Supplementary figures and images for: Impact of differential DNA methylation on transgene expression in cotton (Gossypium hirsutum L.) events generated by targeted sequence insertion
Source: Plant Biotechnol J. 2019 Jan 19;17(7):1236–47. doi: 10.1111/pbi.13049 (PMC6576080; doi:10.1111/pbi.13049)

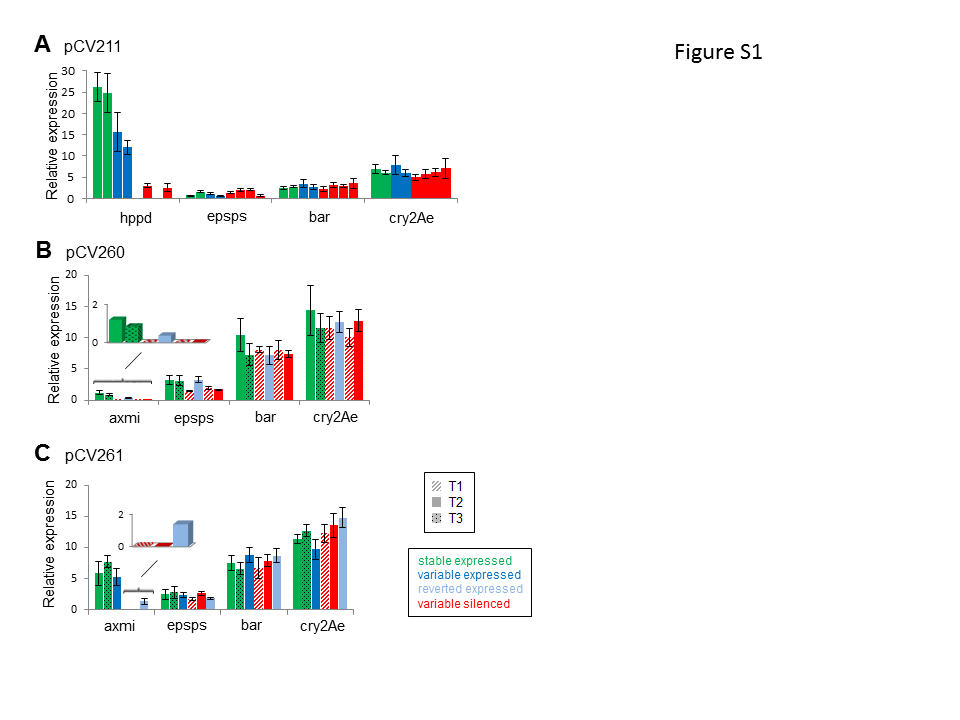

Supplement: Supplementary file 1 — Figure S1 RT‐qPCR in GOI stable expressing, variable expressing and silenced pCV211, pCV260, and pCV261 plants. [file PBI-17-1236-s005.tif]

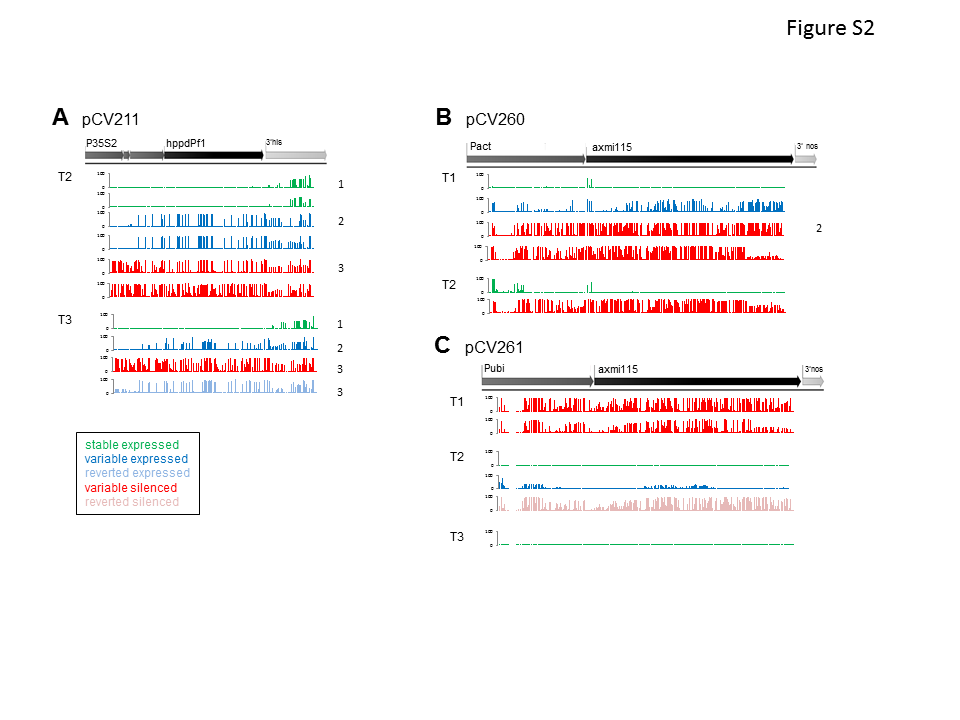

Supplement: Supplementary file 2 — Figure S2 Targeted bisulfite sequencing. [file PBI-17-1236-s004.tif]

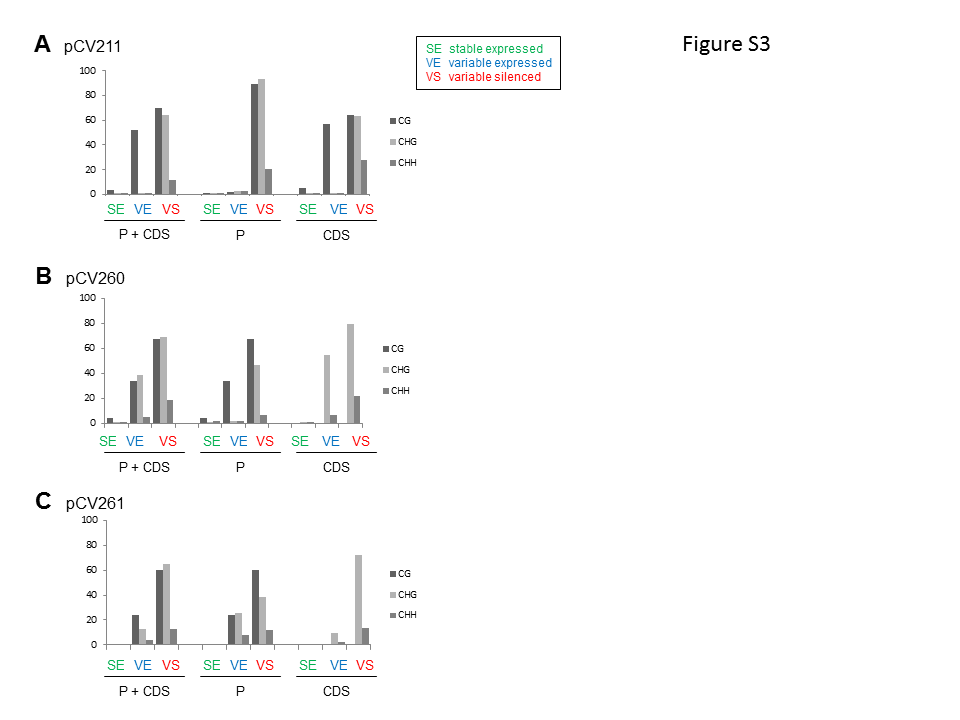

Supplement: Supplementary file 3 — Figure S3 Different methylation contexts invoke unstable or silenced expression in different TSI events. [file PBI-17-1236-s008.tif]

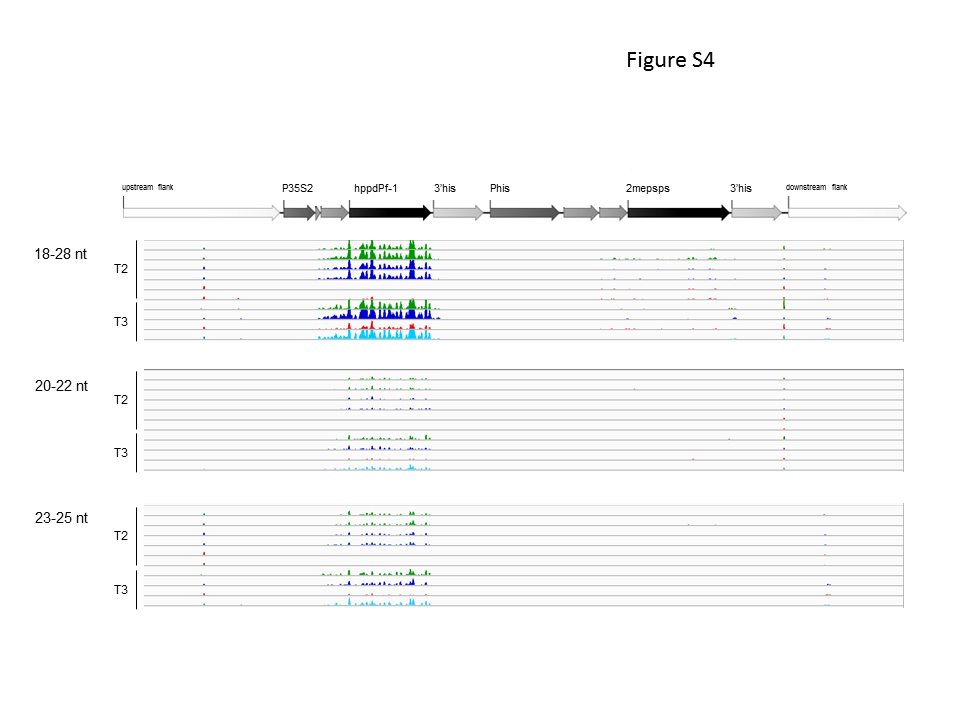

Supplement: Supplementary file 4 — Figure S4 Results sRNA sequencing from pCV211 TSI plants. [file PBI-17-1236-s007.tif]

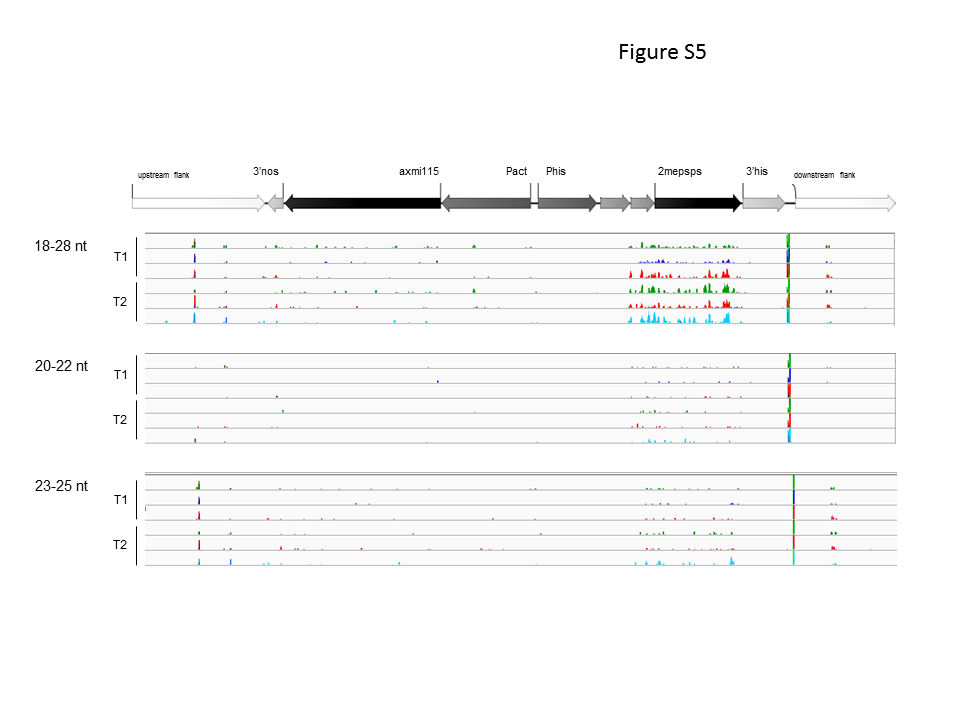

Supplement: Supplementary file 5 — Figure S5 Results sRNA sequencing from pCV260 TSI plants. [file PBI-17-1236-s001.tif]

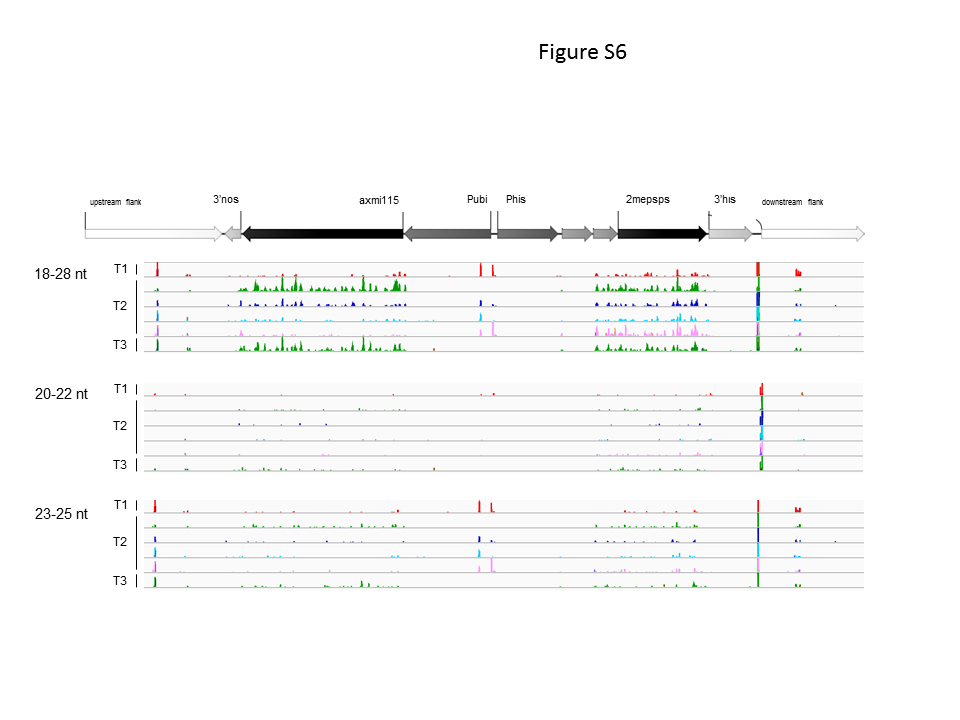

Supplement: Supplementary file 6 — Figure S6 Results sRNA sequencing from pCV261 TSI plants. [file PBI-17-1236-s002.tif]
