# Supplementary material for: Impact of differential DNA methylation on transgene expression in cotton (Gossypium hirsutum L.) events generated by targeted sequence insertion
Source: Plant Biotechnol J. 2019 Jan 19;17(7):1236–47. doi: 10.1111/pbi.13049 (PMC6576080; doi:10.1111/pbi.13049)
Supplement: Supplementary file 8 — Supplementary Legends [file PBI-17-1236-s006.docx]

**Legends supporting information**

**Figure S1. RT-qPCR in GOI stable expressing, variable expressing and silenced pCV211, pCV260, and pCV261 plants.**

RT-qPCR of *hppd*/*axmi*, 2m*epsps*, *bar* and *cry2Ae* transcript accumulation in plants from different TSI events over generations (T1, T2, T3). The T1, T2, T3 generations are indicated by the pattern fill in the bar: diagonal stripes for T1; solid fill for T2; dots for T3. Expression of *hppd/axmi115*, 2m*epsp*, *bar*, *cry2Ae* relative to the reference gene *Gh-pp2a* is shown. RT-qPCR analysis is performed on a subset of plants as indicated in the RT-qPCR column of Table S4. (A) pCV211 plants from event G4GH9000-023, (B) pCV260 plants from events G4GH9029-065 and G4GH9044-025 and (C) pCV261 plants from event G4GH9057-110 and G4GH9041-166. Green, “stable” expressed; dark blue, “variable” expressed; red, “variable” silenced; pale blue, “reverted” expressed. pCV211 donor DNA contains a 2m*epsps*/*hppd* expression cassette; pCV260 and pCV261 represent donor DNA with a 2m*epsps*/*axmi115* expression cassette (Table S1). Each bar shows the relative expression of a single plant. Plants are shown in the same order for *axmi*, 2m*epsps*, *bar* and *cry2Ae*.

**Figure S2. Targeted bisulfite sequencing.**

Targeted bisulfite sequencing on additional progeny plants of stable expressing (green), variable expressing (blue) and silenced (red) plants over different generations (T1, T2, T3). Mean methylation density per cytosine in all contexts is plotted on a 0-100% scale. (A) pCV211 G4GH9000-023_1 and G4GH9000-023_2 plants. (B) pCV260 G4GH9029-065_1, G4GH9044-025_1 and G4GH9044-025_2 plants. (C) pCV261 G4GH9057-110_1, G4GH9057-110_2 and G4GH9041-166_2 plants. pCV211 donor DNA contains a 2m*epsps*/*hppd* expression cassette; pCV260 and pCV261 represent donor DNA with a 2m*epsps*/*axmi115* expression cassette (Table S1). The numbers to the right in Figure S2 A refer to which plant gave rise to which progeny plant. Plants with the same number refer to a progenitor/progeny pair. For the pCV260 and pCV261 events (Figure S2 B, C), BS-seq on T2 and T3 generations was performed on plants with different expression classes from which the expression pattern was known but not the methylation pattern (BS-seq data) for their progenitors.

**Figure S3. Different methylation contexts invoke unstable or silenced expression in different TSI events.**

Context specific methylation percentages in (A) pCV211, (B) pCV260, and (C) pCV261 donor DNA TSI events as determined by bisulfite methylation sequencing. Methylation percentages in the complete GOI (P + CDS), promoter (P) and coding sequence (CDS) are shown. Plants analyzed originate from the same events as in Figure 5 (see Table S4). Green, “stable” expressed; dark blue, “variable” expressed; red, “variable” silenced. pCV211 donor DNA contains a 2m*epsps*/*hppd* expression cassette; pCV260 and pCV261 represent donor DNA with a 2m*epsps*/*axmi115* expression cassette (Table S1).

**Figure S4. Results sRNA sequencing from pCV211 TSI plants.**

Mapping of the 18-28 nt, 20-22 nt and 23-25 nt sRNA sequencing reads to the complete transgene insert sequence. The y axes are scaled the same per sample, 0 to 150 mapped reads are visualized. Reads were normalized per 10 million of genome-matched (including the transgene sequence) 18-to-28 nucleotide sequences. Green, “stable” expressed; dark blue, “variable” expressed; red, “variable” silenced; pale blue, “reverted” expressed; and pink, “reverted” silenced plants. pCV211 donor DNA contains a 2m*epsps*/*hppd* expression cassette.

**Figure S5. Results sRNA sequencing from pCV260 TSI plants.**

Mapping of the 18-28 nt, 20-22 nt and 23-25 nt sRNA sequencing reads to the complete transgene insert sequence. The y axes are scaled the same per sample, 0 to 50 mapped reads are visualized. Reads were normalized per 10 million of genome-matched (including the transgene sequence) 18-to-28 nucleotide sequences. Green, “stable” expressed; dark blue, “variable” expressed; red, “variable” silenced; pale blue, “reverted” expressed; and pink, “reverted” silenced plants. pCV260 donor DNA contains a *2mepsps/axmi* expression cassette.

**Figure S6. Results sRNA sequencing from pCV261 TSI plants.**

Mapping of the 18-28 nt, 20-22 nt and 23-25 nt sRNA sequencing reads to the complete transgene insert sequence. The y axes are scaled the same per sample, 0 to 50 mapped reads are visualized. Reads were normalized per 10 million of genome-matched (including the transgene sequence) 18-to-28 nucleotide sequences. Green, “stable” expressed; dark blue, “variable” expressed; red, “variable” silenced; pale blue, “reverted” expressed; and pink, “reverted” silenced plants. pCV261 donor DNA contains a *2mepsps/axmi* expression cassette.

**Table S1. List of donor DNAs and obtained TSI frequencies.**

**Table S2. Overview of ELISA on T0 plants.**

**Table S3. Summary of targeted DNA sequencing results.**

**Table S4. List of all TSI event plants/progeny analyzed.**

**Table S5. Bismark targeted DNA methylation sequencing reports.**
